# Chemical Seed Priming: Molecules and Mechanisms for Enhancing Plant Germination, Growth, and Stress Tolerance

**DOI:** 10.3390/cimb47030177

**Published:** 2025-03-07

**Authors:** Mason T. MacDonald, Vijaya R. Mohan

**Affiliations:** Department of Plant, Food and Environmental Sciences, Faculty of Agriculture, Dalhousie University, Bible Hill, NS B2N 5E3, Canada; vj797230@dal.ca

**Keywords:** antioxidants, ascorbic acid, biostimulant, epigenetic, nanoparticles, seed preconditioning, plant stress memory

## Abstract

Food security is one of the world’s top challenges, specifically considering global issues like climate change. Seed priming is one strategy to improve crop production, typically via increased germination, yields, and/or stress tolerance. Hydropriming, or soaking seeds in water only, is the simplest form of seed priming. However, the addition of certain seed priming agents has resulted in a variety of modified strategies, including osmopriming, halopriming, hormonal priming, PGR priming, nutripriming, and others. Most current research has focused on hormonal and nutripriming. This review will focus on the specific compounds that have been used most often over the past 3 years and the physiological effects that they have had on crops. Over half of recent research has focused on four compounds: (1) salicylic acid, (2) zinc, (3) gibberellic acid, and (4) potassium nitrate. One of the most interesting characteristics of all chemical seed priming agents is that they are exposed only to seeds yet confer benefits throughout plant development. In some cases, such benefits have been passed to subsequent generations, suggesting an epigenetic effect, which is supported by observed changes in DNA methylation and histone modification. This review will summarize the current state of knowledge on molecular changes and physiological mechanisms associated with chemical seed priming agents and discuss avenues for future research.

## 1. Introduction

Climate change is posing a significant challenge to agriculture and food security. The average global temperature has increased by 1.41 °C since the preindustrial period [1], which has contributed to an increased frequency of extreme weather events [2,3,4]. Such environmental changes are adversely affecting the yields of many crops [5,6,7]. For example, wheat yields are expected to decrease by 4–6% for every degree increase in global temperature [8,9]. A meta-analysis of future wheat yields predicted decreases of 18% and 31% due to temperature and drought stress, respectively [10]. The future risk to food security posed by climate change necessitates the exploration of strategies to increase growth and stress tolerance.

**Figure 1:**
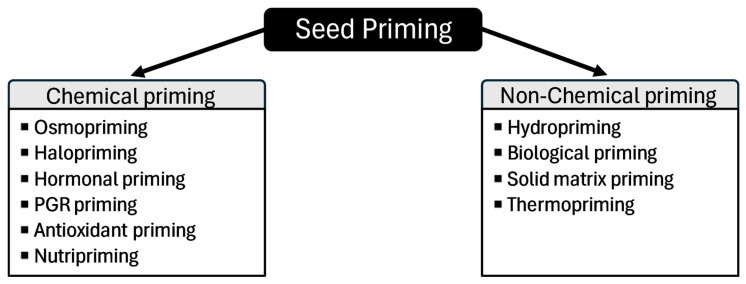
List of chemical and non-chemical seed priming methods.

Seed priming has high potential to promote crop resilience to climate change. At its most basic level, seed priming involves soaking seeds in water to promote germination [11]. However, seed priming technologies have improved to include a variety of chemical and non-chemical agents (Figure 1). These seed priming agents (SPAs) are intended to promote germination, growth, and/or stress tolerance in developing plants [12,13,14]. The focus of this review is on chemical seed priming as opposed to non-chemical priming. Many chemicals have been used as SPAs, such as ascorbic acid [12], salicylic acid (SA) [15], glycinebetaine [15], benzimidazoles [16], pyroligneous acid [17], and other naturally occurring antioxidants [18]. Such chemical SPAs can be broadly classified as osmotic chemicals, salts, hormones, plant growth regulators, antioxidants, and nutrients [13,19,20].

One of the primary reasons for seed priming is to improve seed germination. Seed germination is a crucial part of a plant’s life cycle and is particularly susceptible to environmental stress [21]. Priming seeds improves germination, emergence, field uniformity, and seed vigor [22,23,24]. Specific details of physiological and molecular pathways continue to be discovered, but priming is known to activate critical metabolic processes within a seed, such as enzyme activation or resource mobilization, prior to radicle emergence [25].

Perhaps the most curious aspect of seed priming is that it triggers beneficial effects that persist in developing plants. Specific effects vary somewhat depending on the SPA but often include improvements in photosynthesis, nutrient content, oxidative stress regulation, and hormonal dynamics [13]. For example, broccoli seeds primed with 10 ppm ascorbic acid resulted in plants with an 84% increase in photosynthesis under irrigated conditions [12]. Those broccoli seedlings also exhibited improved drought tolerance when water was withheld, maintaining photosynthesis at similar rates to irrigated non-primed seedlings [12]. Improvements in photosynthesis and stress tolerance have inevitably led to increased yields in several studies [12,26,27], though improvements in yields have not always occurred in outdoor field trials [28]. Still, the possibility of increasing yields through inexpensive seed preconditioning techniques has justified research into the modes of action of SPAs. The objective of this review is to describe seed priming techniques, with an emphasis on the common chemicals used. This review will identify SPAs that have been studied most often in the past 3 years. This review will also describe what is known about chemical SPA molecular biology (with an emphasis on “omics” approaches), gaps in our understanding, and areas needed for future research.

## 2. Seed Priming Techniques

Seed priming is a method where seeds imbibe water, typically in the presence of a priming agent or other specifically imposed conditions [29,30]. The method is relatively short in duration, allowing a seed to reach a pre-germinative metabolism but stops before reaching full germination [31]. Germination occurs in three phases. Phase 1 involves rapid imbibition of water due to differences in water potential of a seed and its surroundings, phase 2 begins with the activation of seed metabolic processes, and phase 3 involves rapid uptake of water culminating in radicle protrusion [32]. Seed priming lasts only long enough to enter phase 2, ending before reaching phase 3 [33]. When used effectively, seed priming promotes germination or stress mitigation in cultivated species [34]. Although the priming duration is short, benefits can last for the entire life cycle of the plant [34] or possibly be passed to offspring [16].

Seed priming techniques can be broadly classified as chemical and non-chemical priming. This review will focus on the SPAs associated with chemical priming. However, non-chemical priming will be discussed briefly as they include some of the original priming methods before various SPAs were identified.

### 2.1. Non-Chemical Priming

Hydropriming is the simplest form of seed priming, considered to be the pragmatic approach because it is cost-effective, safe, and eco-friendly [35]. Hydroprimed seeds are simply soaked in water, which allows seeds to go through their usual germination phases [20]. It is the duration of priming that is the critical factor for hydropriming. Seeds should only be primed long enough to initiate normal metabolic processes, which makes it difficult to create a universal procedure [35]. For example, optimum response is achieved with 96 h of priming in onions [36], but can be achieved in as little as 7 h in pinto beans [37].

There are several other non-chemical priming methods. Thermopriming exposes seeds to high or low temperatures [38,39], biological priming combines imbibition with microbial inoculation [19,40], and solid matrix priming treats seeds with a known combination of water and solid material [20,41]. There has been increasing interest in other non-chemical priming techniques, such as magnetopriming, which involves passing seeds through a magnetic field [42,43]. A review by Farooq et al. [29] elaborates on the potential of non-chemical priming methods.

### 2.2. Chemical Priming

If hydropriming is the incubation of seeds with water alone, then chemical priming offers a logical next step by incubating seeds with virtually any compound (often dissolved or diluted in water) [44]. Many substances used for chemical priming are naturally occurring, such as plant extracts, chitosan, phytohormones, plant growth regulators, sugars, or polyamines [44]. The classification of chemical SPAs varies slightly from study to study, but a general classification scheme can be seen in Figure 1.

**Figure 2:**
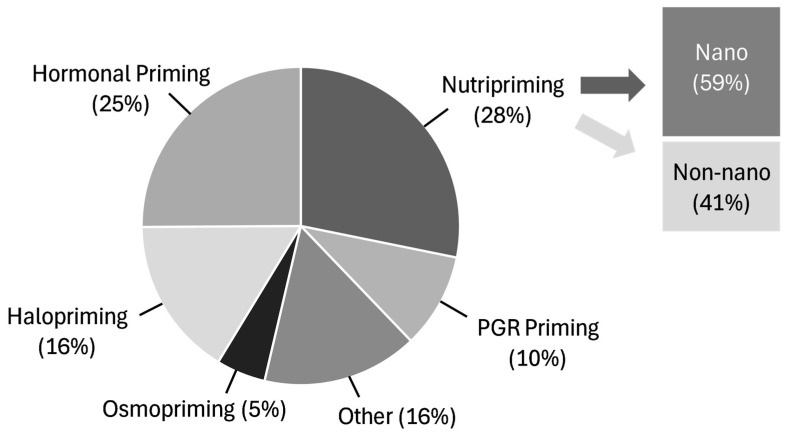
Distribution of different chemical SPAs used as determined by 153 peer-reviewed papers over the past 3 years. The other category includes amino acids, chitosan, plant extracts, wood distillates, and antioxidants, which do not easily fit into other SPA classes. The full list of references from which this figure was created can be found in Supplementary Materials File S1.

Chemical priming works by exposing seeds to stimuli beyond simple imbibition [45,46]. One of the earlier examples of chemical priming involved priming cress and lettuce seeds in sea water (an early example of halopriming) to improve germination [47,48]. More modern tests use nutrient solutions to prime tomato seeds [49] and kickstarted studies on many different potential SPAs [14,20,21]. In the past 3 years alone, there have been at least 64 different SPAs included in peer-reviewed studies (Supplementary Materials File S1). Most of the studies focused on nutripriming and plant growth regulator (PGR) priming, followed by halopriming and hormone priming (Figure 2). The objective of this section is to briefly describe the types of chemical priming most often studied before focusing on specific SPAs.

#### 2.2.1. Osmopriming

Osmopriming is the general term for soaking seeds in a solution with low water potential [20]. By that definition, most chemical SPAs could be considered osmoprimers. However, osmopriming more often refers to sugar solutions such as polyethylene glycol (PEG), glycerol, sorbitol, or mannitol [20]. The most common osmoprimer studied in the past 3 years is PEG, which agrees with the previous literature [50].

Osmopriming introduces several more advantages than hydropriming alone. First, osmopriming results in earlier germination and seedling emergence [14]. Second, effects from osmopriming tend to continue throughout plant development long after germination [50,51]. Finally, osmopriming offers a better improvement in stress tolerance [52,53].

#### 2.2.2. Halopriming

Halopriming is performed by soaking pre-germinated seeds in inorganic salt solutions [54]. Several salts have been used historically, such as NaCl, Na_2_CO_3_, KCl, KNO_3_, CaCl_2_, and CaSO_4_ [54]. However, KNO_3_ and CaCl_2_ have been the most studied over the past 3 years, being included in 12% and 9% of all seed priming studies since 2022 (Table 1).

Water potential is greatly reduced during halopriming, which is comparable to osmopriming. However, there are two key distinctions between halo- and osmopriming. The first is that osmotic adjustment is facilitated by adding a salt usually to distilled water [20]. The second is that the cation of the salt is often an essential element for plant growth, such as Ca^2+^ or K^+^ [54]. Still, there remains some overlap in the literature where halopriming agents will be described as osmopriming agents [14,21]. It may be best to consider halopriming as a subsect of osmopriming.

#### 2.2.3. Hormonal Priming

Hormonal priming, sometimes referred to as hormopriming, refers to a strategy of soaking seeds in specific concentrations of phytohormones to elicit a response [55]. Phytohormones play significant roles in signaling pathways for normal physiological function but are also key mediators for stress responses [56,57,58]. The “classical five” phytohormones are gibberellic acid (GA), ethylene, auxin, cytokinins, and abscisic acid (ABA) [59]. Other compounds including jasmonates, brassinosteroids, and SA have since been regularly discussed as phytohormones [60,61,62]. These eight compounds are classified as hormonal primers for the purposes of this review. Other compounds, such as melatonin, are sometimes referred to as phytohormones [63] but currently remain classified as PGRs in this review.

Phytohormones may been separated into two categories. The first is hormones associated with growth promotion such as auxins, cytokinins, gibberellins, and brassinosteroids [62]. The second is hormones associated with stress tolerance such as ABA, jasmonic acid, and ethylene [64]. SA and GA are easily the most popular SPAs used for hormonal priming over the past 3 years, which together account for almost one-third of sampled SPA studies over the past 3 years (Table 1).

#### 2.2.4. Plant Growth Regulator Priming

PGR priming is very similar to hormonal priming, to the extent that some papers use the terms interchangeably. Like other priming treatments, PGR priming involves soaking seeds in low concentrations of PGRs to elicit a positive response [65]. PGRs tend to be organic compounds that play vital roles in growth, expansion, and yield of plants even when present in small amounts [65]. PGRs may act as chemical messengers to regulate a range of cellular processes or help to coordinate complex signal transduction pathways during abiotic stress [66].

The phytohormones listed in Section 2.2.3 meet the definition of PGRs. But in much the same manner that not all fingers are thumbs, not all PGRs are phytohormones. Compounds such as tocopherols, polyamines, quaternary ammonium compounds, proline, trehalose, ascorbic acid, melatonin, and strigolactones are all considered PGRs, though they are not conventionally discussed as phytohormones [66,67]. Of these non-hormonal PGRs, melatonin and ascorbic acid have been studied most often as an SPA (Table 1).

#### 2.2.5. Nutripriming

Nutripriming is performed by soaking seeds in solutions containing micro- or macronutrients [68]. Nutripriming combines the biochemical effect from hydropriming with the nutritional effect of whichever nutrient is used. Many nutrients have been used, either individually or in combinations [69]. For example, Mg, Zn, and B have all increased germination and yield of several crops [70,71,72]. However, Zn and Se have been studied most often and account for a quarter of all chemical SPA studies over the past 3 years (Table 1).

#### 2.2.6. Other Chemical Priming

There are many other compounds used for seed priming that do not fall neatly into the more popular classifications. Chitosan, a bioactive polysaccharide molecule usually obtained from crustacean shells [73,74], is the most studied compound in the past 3 years that does not belong in the above classifications (Table 1). But turmeric [75], biochar [76], fluridone [77], essential oils [78], pyroligneous acid [79], H_2_O_2_ [80], and humic acid [81] have all demonstrated beneficial effects as SPAs in recent years. The use of H_2_O_2_ has been called redox priming in some papers, using direct exposure to free radicals to promote abiotic stress defense [80].

The use of plant extracts as SPAs is a research area that continues to develop. Plant extracts can contain a cocktail of phytochemicals, which are likely a blend of multiple classifications of SPA. Extracts from several different plants have been used. For example, Dawoud et al. [82] used extracts from pomegranate peels to prime cabbage seeds to improve growth characteristics. Meanwhile, El Sayed et al. [83] used extracts from cypress leaves to promote photosynthesis, growth, and antioxidant defense in zucchini. It is likely that there are many other plant extracts that could elicit a positive response.

## 3. Commonly Used SPAs and Their Effects

### 3.1. Salicylic Acid

SA (2-droxybenzoic acid) is potent phenolic signaling molecule and is widely involved in many physiological processes, such as germination, reproductive and vegetative growth, fruit ripening, and photosynthesis [84]. Further, SA is also involved in abiotic stress tolerance [85,86]. The multifunctional role of SA in plant physiology made it a desirable candidate for seed priming.

There are mixed results with respect to salicylic acid and germination. Several recent studies report an improvement in seed germination after SA priming [81,85,87,88,89,90], but not all had an experimental design capable of distinguishing whether SA offered any improvement beyond water alone. For instance, Ceritoglu et al. [90] found a significant increase in germination percentage and a decrease in germination time in lentils compared to an unprimed control but with no comparison to hydropriming. Meanwhile, Alam et al. [85] found no difference in the germination of cucumbers after priming with a 0.005% SA solution. There was also no improvement in the germination of rice seeds after priming with 10 mg L^−1^ or 20 mg L^−1^ SA versus hydropriming alone [89]. Conversely, priming with 0.2 mM SA increased germination in eggplant and sweet peppers [81]. Hydroprimed eggplant and sweet pepper seeds germinated at approximately 75 and 70%, respectively. Meanwhile, SA priming improved germination to approximately 92% in eggplant and 85% in sweet pepper [81]. Improvements in growth and yield due to SA seed priming have been much more consistent.

### 3.2. Zinc

Zn is a vital micronutrient for plants, and it plays a key role in essential cellular functions, including metabolism, physiological activities, and the regulation of ion homeostasis [91,92]. Zn plays an important role in photosynthesis by acting as a key component in chlorophyll synthesis [93]. It also plays a significant role in alleviating abiotic stresses such as salinity, drought, and extreme temperatures by minimizing oxidative damage [94]. This versatile nature of zinc in plant physiology makes it a potential seed priming agent.

Positive results have been observed in germination through Zn seed priming. Several recent studies have reported improved germination in rice, wheat, and spinach following Zn seed priming [71,95,96]. However, a study on corn indicated that both Zn and hydropriming enhanced germination [97]. Additionally, Zn seed priming was utilized in spinach to improve its germination rate and resistance to low-temperature stress during germination and early seedling development [95]. Zn seed priming has been shown to significantly improve crop performance across various plant species, including wheat, maize, and pea, particularly in Zn-deficient soils. In wheat, priming with 0.5 M ZnSO_4_ for 12 h enhanced seedling growth and establishment [96]. Similarly, maize seed priming with a 1% Zn solution for 16 h resulted in increased seedling weight, height, and Zn content, leading to a 27% increase in grain yield compared to non-primed seeds on calcareous, Zn-deficient soils in Pakistan [98]. In pea, priming with 1% ZnSO_4_ for 12 h improved yield-related traits such as pod length, number of pods per plant, and number of grains per pod, thereby enhancing overall green pea yield compared to hydroprimed seeds [99]. Overall, Zn priming treatments, particularly at optimal concentrations and durations, significantly boost seedling growth, yield attributes, and crop yield, making them a promising cost-effective agronomic tool for improving productivity.

### 3.3. Gibberellic Acid

Gibberellic acid (GA_3_) is a vital plant hormone influencing various stages of the plant life cycle [100]. Its functions include regulating seed germination, leaf expansion, stem elongation, flower and trichome initiation, source–sink dynamics and the development of flowers, fruits, and seed [100,101]. GA_3_ is categorized among the five classical plant hormones, alongside auxins, cytokinins, ABA, and ethylene, each of which influences specific physiological traits in plants [59]. GA_3_ is particularly notable for its role in controlling plant stature and alleviating seed dormancy [102,103]. At the molecular level, GA_3_ promotes plant elongation by regulating cell growth processes [102]. These multifaceted roles of GA_3_ in plant physiology make it an excellent seed priming agent.

GA_3_ priming has been widely studied across various crops, showing significant improvements in germination and growth-related traits. For instance, it enhanced seed germination rates, plant height, and overall biomass in *Leymus chinensis* [103]. In maize, GA_3_ priming increased germination percentage, reduced mean germination time, and improved seedling vigor under low-water-potential conditions [104]. Similarly, in tomato, optimal results were observed with GA_3_ priming at specific concentrations, enhancing germination rates, shoot and root lengths, and seed vigor [105].

Despite these findings, limited studies have evaluated GA_3_ priming effects through maturity or yield. Notably, GA_3_ priming improved growth traits under severe drought in rapeseed [106]. Under salinity stress, GA_3_ priming enhanced germination percentage, reduced germination time, and improved shoot and root length in crops like *Zea mays* L., *Pisum sativum*, and *Lathyrus sativus* [107]. Furthermore, GA_3_ priming enhanced germination, seedling dry weight, and antioxidant enzyme activity while reducing membrane damage in alfalfa [108]. In rice, it improved growth, chlorophyll content, and antioxidant capacity, alleviating salt-induced physiological stress [109].

The consistent improvements in germination and stress tolerance due to GA_3_ priming highlight its potential as an effective strategy for enhancing plant resilience and productivity under challenging conditions.

### 3.4. Potassium Nitrate

Potassium nitrate (KNO_3_) is an ionic salt composed of potassium ions (K⁺) and nitrate ions (NO_3_^−^), and is widely used in seed priming due to its role as a major essential plant nutrient [110]. Studies have demonstrated its effectiveness in improving seed germination, seedling growth, and yield across various crops. For instance, KNO_3_ priming enhanced germination percentage, germination index, and reduced mean germination time in holy basil seeds [110], while rice seeds showed improved germination percentage, speed, and uniformity compared to untreated seeds [111]. Similarly, soybean seeds primed with KNO_3_ exhibited increased radicle and plumule length, seedling dry weight, plant height, leaf area, and overall plant dry weight [112]. Tomato seeds also benefited significantly, with improved final emergence percentage, mean emergence time, and physiological attributes, as well as higher levels of total soluble sugars and phenolics [113]. These benefits extended to chickpea seeds, which displayed enhanced germination and seedling vigor following KNO_3_ seed priming [114]. Moreover, rice seeds treated with KNO_3_ experienced a 7% increase in yield due to improved germination and growth parameters [115].

In addition to its role in enhancing germination and yield, KNO_3_ priming has proven effective in improving stress tolerance in crops. Soybean seeds primed with KNO_3_ showed better germination, emergence traits, and seedling growth under salinity stress [112]. Likewise, cantaloupe seeds demonstrated enhanced germination, growth, and fruit yield under drought stress when primed with KNO_3_ [116]. Furthermore, carrot seeds experienced improved germination, seedling growth, and root biomass under high-temperature stress following KNO_3_ seed priming [96]. These findings collectively highlight the versatility and efficacy of KNO_3_ in seed priming, not only for enhancing germination and crop performance under optimal conditions but also for mitigating the adverse effects of abiotic stresses. The consistent results across diverse crops and environmental conditions make KNO_3_ a valuable tool for improving agricultural productivity and sustainability.

### 3.5. Selenium

Se is an essential micronutrient for plant growth [117], playing a pivotal role in various metabolic processes through the synthesis of selenoenzymes. These selenoenzymes act as antioxidants, mitigating oxidative stress by detoxifying reactive oxygen species (ROS) [118]. However, while beneficial in trace amounts, excessive Se can adversely affect plant physiological and biochemical activities [119].

Seed priming with Se has demonstrated notable improvements in germination and stress resilience across various crops. For instance, Se-primed turnip (*Brassica rapa* L.) seeds showed enhanced germination, photosynthetic content, and seedling biomass by 48%, 56%, and 51%, respectively [120]. In Basmati rice, Se priming increased seedling emergence, chlorophyll content, and antioxidant activity compared to untreated and hydroprimed controls [121]. Under stress conditions, Se priming effectively mitigated oxidative stress in turnip exposed to salinity by upregulating antioxidant genes and reducing ROS, malondialdehyde, and proline levels [120]. Similarly, Se priming improved root length, biomass, and drought tolerance in wheat while increasing sugars and amino acids [122]. Additionally, Se priming boosted germination, growth, and dry weight in rice under flooding stress [123] and improved stress tolerance in quinoa under drought [124]. These findings highlight Se’s dual role in enhancing plant growth and mitigating abiotic stresses, making it a valuable agent for seed priming.

## 4. SPA Modes of Action

### 4.1. Overview

One of the most curious characteristics of seed priming is that short-term exposure to SPAs can induce long-term effects in developing plants. For example, broccoli seeds exposed to ascorbic acid for 24 h had significantly improved growth and photosynthesis in 8-week-old seedlings [12,125]. Further, the benefits of seed priming have been observed in the progeny of primed plants [16,126]. Seed priming must therefore induce changes to DNA and protein expression [127,128].

SPAs cause multiple changes at the DNA level. First, seed priming triggers DNA replication, causing an increase in total genomic DNA [129,130]. Second, seed priming activates genes associated with DNA repair [131]. For example, hydropriming activated *GTF II H2*, *MMZ3*/*UEV 1C*, *RAD3*, *Rec A-like 1*, *RAD54*, *U DNA glycosylase*, and *KU 80*, which are all associated with DNA repair and subsequent seed germination [131]. Third, seed priming activates genes associated with a variety of other physiological characteristics, such as enzyme synthesis [33,132], ATP [133,134], phytohormones [134,135], and antioxidants [125,135,136] (Table 2).

**Figure 3:**
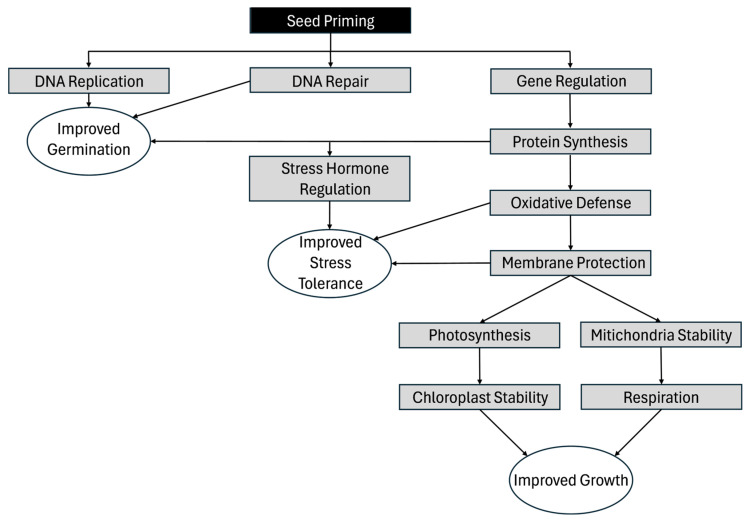
Modes of action for SPAs to achieve their three major purposes (improved germination, stress tolerance, and growth).

Improvement in antioxidant defense systems is one of the more widely reported physiological changes. SPAs activate antioxidant systems by increasing the activities of superoxide dismutase, peroxidase, and catalase while enhancing the accumulation of glutathione and free proline to mitigate oxidative damage [85,122,143]. These agents optimize photosynthetic processes by protecting chlorophyll and photosynthetic machinery, which causes increasing carotenoids and chlorophyll content, which help maintain energy balance and reduce ROS generation [144]. Equally importantly, oxidative protection also helps protect the mitochondria [133,145], ensuring cellular respiration can continue to generate ATP. Stress hormone modulation, particularly ABA signaling, stimulates carotenoid biosynthesis and stomatal closure, thereby improving water-use efficiency [135]. Additionally, seed priming improves resource allocation by optimizing root-to-shoot ratios, nutrient mobilization, and carbon fixation, leading to enhanced biomass accumulation [83,138,139,140,141]. Such changes enrich phytochemical profiles by increasing phenolics, flavonoids, and other bioactive compounds that contribute to stress resilience and nutritional quality [137,138,146]. By bolstering antioxidant defenses, enhancing photosynthetic efficiency, and improving nutrient utilization, SPAs prepare plants to withstand abiotic stresses and improve overall productivity, as shown in Figure 3.

### 4.2. Transcriptomic and Translatomic Changes

Changes during transcription are one of the earliest effects of seed priming [147]. CaCl_2_ priming in barley seeds induced drought tolerance [148]. Transcriptome analysis of those primed barley seeds identified *ERF*/*AP2*, *C2C2-Dof*, and *bHLH* transcription factors associated with drought tolerance [148]. Similarly, a study in seed primed rice identified 27 upregulated transcription factors and 5 downregulated transcription factors [149]. These transcription factors were associated with the regulation of root growth, phytohormone synthesis, and other established physiological benefits of seed priming. Similarly, Se- and SA-primed seeds analyzed 2371 and 2405 transcripts, respectively, identifying several associated with the regulation of secondary metabolism, plant development, and cell transport [150].

Examination of a plant translatome has also been used to further understand the complex changes associated with seed priming. The translatome helps identify the various regulatory events that modulate protein synthesis [151]. For example, internal ribosome entry site *trans*-acting factors play a key role in seed germination [152]. One such factor, *EBP1*, was found to over-accumulate in hydroprimed sugarbeet seeds [153]. *EBP1* is broadly described to drive plant growth, specifically through root growth. Silencing *EBP1* reduces root growth, while overexpressing *EBP1* promotes root growth [154]. However, de novo protein synthesis increased the synthesis of ROS scavenging enzymes, providing a link to priming-induced stress tolerance [155].

### 4.3. Proteomic Changes

Seed priming induces many changes in protein accumulation. Proteomic analyses of melatonin-primed oat seeds and ascorbate primed wheat seeds uncovered 201 and 83 differentially expressed proteins (DEPs), respectively [156,157]. Halopriming of wheat uncovered 21 DEPs, 17 of which were upregulated [158]. The number and specific DEPs induced through priming seem to vary based on priming agent and plant species, but they often share similar functions. Some are related specifically to cell division [159], but other common functions include RNA repair, antioxidant protection, carbohydrate metabolism, photosynthesis, and electron transfer [156,157]. One of the most commons responses is an increase in ROS scavenging enzymes, such as catalase, superoxide dismutase, or ascorbate peroxidase [84,145,160].

### 4.4. Metabolomic Changes

Metabolites are often subdivided into primary and secondary classes. Primary metabolites include compounds like amino acids, sugars, nucleotides, and organic acids; secondary metabolites include compounds like phenolics, flavonoids, and others [161]. Changes in both primary and secondary metabolites are often reported in the literature.

Sugars and amino acids are often the most commonly reported changes in primary metabolites due to seed priming. Multiple methods of seed priming have increased α-amylase activity and, consequently, total soluble sugars [162,163]. Increased soluble sugars could contribute to noted improvements in respiration, which is essential for improved growth [133]. The accumulation of amino acids also offers protection for plants. Se-seed-primed wheat had increased alanine, leucine, glycine, cerine, valine, cysteine, and arginine [164], while AsA-seed-primed corn had increased serine, tyrosine, alanine, valine, glutamate, arginine, proline, aspartate, lysine, and isoleucine [165].

Phenolics and flavonoids are the most reported secondary metabolites associated with seed priming. Various crops have shown higher phenolics and flavonoids due to multiple seed priming agents, as reviewed by Jatana et al. [161]. Conversely, broccoli primed with AsA had higher flavonoids and carotenoids with no impact on total phenolics [125]. In all cases, these secondary metabolites are typically of interest due to their antioxidant activity [166] and the role that they could play under stress.

### 4.5. Epigenetics and Plant Memory

The epigenetic effect of SPAs has often been associated with “stress-induced memory”, where exposure to stress preconditions a plant to respond to future stress more efficiently [34]. In essence, a plant “remembers” its encounter with stress, and this memory could be passed to future generations. The concept of plant memory is not new and has been observed in many species [167,168,169]. However, the idea that this memory may be implanted by SPAs has yet to be fully explored. It has been proposed that SPAs may impose a minor or non-lethal stress within the seed that creates the epigenetic effect [34], and chromatin-dependent regulation is considered a key mechanism [170]. Epigenetic changes in chromatin structure are made possible through processes such as DNA methylation and histone modifications [171,172], which have been shown to occur in some studies. For instance, soybean seeds primed with epethon had an altered chromatin architecture, decreased DNA methyl transferase activity, and decreased DNA methylation [171]. A second study with epethon in soybean identified increased expression of histone acetyltransferase and decreased histone deacetylase [173]. Seed priming effects of DNA methylation and histone modification are limited and represent an area that should be explored further to better understand SPA epigenetic modes of action.

## 5. Nanoparticle Priming Agents

Nanotechnology in agriculture, particularly in the form of seed nano-priming, has emerged as a transformative approach to addressing critical challenges in global agriculture. With environmental stressors such as climate change, soil contamination, and water scarcity threatening food security, innovative methods are required to ensure sustainable agricultural productivity. Seed nano-priming involves the application of nanoparticles (NPs) under 100 nm to seeds during early growth stages, enhancing germination, seedling vigor, and plant health [137,174]. This technique has garnered significant attention for its potential to mitigate abiotic and biotic stressors and improve crop yields.

ZnO NPs have demonstrated efficacy through seed nano-priming, offering benefits such as enhanced germination rates, increased seedling and plant growth, and improved indicators of plant health when applied during early sowing stages. This approach leverages mechanisms like enhanced nutrient uptake, strengthened antioxidant properties, reduced ROS accumulation, and decreased lipid peroxidation [175]. These benefits are particularly crucial for staple crops, which are essential for addressing the demands of a growing global population and mitigating the decline in crop production caused by climate change and soil contamination [175]. Notably, studies have demonstrated the efficacy of ZnO NPs and Ag NPs in enhancing germination and plant growth in crops like bitter gourd and watermelon [137,176]. ZnO NPs, for instance, improve nutrient uptake, strengthen antioxidant responses, and reduce oxidative damage, while Ag NPs derived from agro-industrial byproducts have been shown to increase germination rates and field yields without compromising fruit quality [137,176].

The variability in the effectiveness of NPs due to differences in preparation methods and structural properties remains a subject of debate. Moreover, concerns regarding the potential toxicity of high NP concentrations in crops like lettuce, wheat, and tomatoes underscore the need for careful optimization and application [177]. There are also emerging innovations such as the integration of endophytes and advanced seed coating technologies that help to maximize the benefits of nano-priming. This study concludes that while nano-priming, with its low-cost potential, offers immense potential for sustainable agriculture, further research is needed to refine nanoparticle engineering and address associated risks, ensuring its practical application in mitigating food security challenges globally.

## 6. Conclusions and Future Directions

Seed priming is undoubtedly a good strategy for the improvement of crop production. It is well established that SPAs induce a variety of beneficial changes in plants, such as improved germination, photosynthesis, respiration, antioxidant defense systems, stress tolerance, and growth. SPAs also represent a generally low-cost technology to enhance crop germination, growth, and stress tolerance. For instance, salicylic acid was the most studied SPA in the past 3 years and was effective in 100% of those studies. The current cost of salicylic from Sigma-Aldrich is CAD 170 for 500 g of salicylic acid. As an example, Alam et al. [87] found 0.75 mM was the most effective concentration for seed priming cucumbers. This means a person could make 1 L of seed priming solution for CAD 0.06, with 1 L being sufficient to prime hundreds of seeds. The exact cost varies based on the specific SPA, but there are several inexpensive SPAs demonstrating high efficacy (e.g., ascorbic acid, chitosan, KNO_3_, CaCl_2_, and others). However, the majority (63%) of seed priming research is being conducted in only three countries: India, China, and Pakistan [34]. There is a need to better extend this knowledge on a global scale.

One challenge moving forward is the need for a comprehensive, open-access database of SPAs. Not every SPA is effective in every plant, and they do not all induce the same type or degree of benefits. For example, ascorbic acid was effective at significantly promoting germination and growth in broccoli seedlings [12,125], but was ineffective in 29% of sampled studies in the past 3 years. There are also distinct differences in effective concentrations. A catalog of SPAs, concentrations, effects, and affected crops would be beneficial.

This review discussed some molecular and physiological changes associated with seed priming, including possible epigenetic changes. Though there were some molecular data to support these changes, the data were not abundant. There is a need to further develop the SPA mode of action. Omics approaches are needed to better identify molecular changes from SPAs with emphasis on exploring other epigenetic changing mechanisms beyond DNA methylation and histone modification. Such studies would not only add to the knowledge of how SPAs work but could also provide insight into the long-term stability of epigenetic changes and association with plant stress memory.

## Figures and Tables

**Table 1:** List of the most studied chemical SPAs from 2022 to 2024 as determined by the frequency of their use in 153 peer-reviewed articles. Efficacy refers to the percentage of reported studies where the SPA demonstrated significantly improved response versus hydropriming. The full list of 153 studies is shown in Supplementary Materials File S1.

Common Primer (% of Studies)	Primer Class	Efficacy (%)	Plants Studied
1. Salicylic acid (18%)	Hormone	100	Peppers, chia, sesame, barley, peas, cotton, rice, wheat, barley, cucumber, lentils, mung bean, corn, canola, cantaloupe, wild sunflower, kidney bean, zucchini, eggplant, cumin, hargel
2. Zn (16%)	Nutri	100	Millet, gourd, corn, mung bean, peas, rice, spinach, chick peas, wheat, sesame, quinoa, corn, rice, peppers, eggplant, safflower, lettuce
3. Gibberellic acid (13%)	Hormone	90	Carrot, tomato, sunflower, rice, legumes, alhagi, okra, cotton, barley, hargel, cucumber, tomato, wheat, cowpea, chia, lettuce
4. KNO_3_ (12%)	Halo	68	Carrot, lettuce, sunflower, rice, wheat, mustard, cotton, tomato, cucumber, chick pea, wild sunflower, corn, cowpea, pepper, chia
5. Se (9%)	Nutri	100	Quinoa, tomato, jalapeno, turnip, bok choy, wheat, sorghum, mustard, rice, canola
6. CaCl_2_ (9%)	Halo	86	Lettuce, barley, mustard, wheat, peas, rice, allium, canola
7. PEG (8%)	Osmo	92	Tomato, wheat, Chinese skullcap, onion, cauliflower, peppers, allium, tomato
8. Chitosan (7%)	Other	100	Ashwagandha, corn, lettuce, mung bean, wheat, clover, cumin
9. Melatonin (5%)	PGR	100	Zinnia, peanuts, halophytes, wheat, corn, rice, triticale
10. Ascorbic acid (5%)	PGR	71	Rice, wheat, molinga, broccoli, stevia

**Table 2:** Summary of some most studied seed priming agents (SPAs), their target crops, and their mode of action.

Seed Priming Agent	Crop	Modes of Action
Selenium	Rice, Wheat	Activates antioxidant enzymes (SOD, POD, CAT), increases chlorophyll content, and enhances stress tolerance [122,125].
Ascorbic Acid	Broccoli	Increases carotenoid content, protects chloroplasts, maintains photosynthetic machinery, and modulates ABA.
Zn Oxide Nanoparticles	Bitter Gourd	Enhances phenolic and flavonoid content, and improves phytochemical profile [137].
Sodium Nitroprusside	Wheat	Activates antioxidant defense, enhances phenolic content, and improves photosynthesis and transpiration [138].
Melatonin	Maize	Reduces membrane permeability, enhances photosynthetic efficiency, and protects chlorophyll [139,140].
Gibberellic Acid	Cucumber, Rapeseed	Increases photosynthesis, transpiration rates, and chlorophyll content [141,142].
Salicylic Acid	Zucchini	Enhances photosynthesis, antioxidant capacity, and chlorophyll content [83].

## Data Availability

No new data were produced in this research.

## Supplementary Materials

The following supporting information can be downloaded at: https://www.mdpi.com/article/10.3390/cimb47030177/s1.
