# Supplementary material for: Chemical Seed Priming: Molecules and Mechanisms for Enhancing Plant Germination, Growth, and Stress Tolerance
Source: Curr Issues Mol Biol. 2025 Mar 7;47(3):177. doi: 10.3390/cimb47030177 (PMC11941364; doi:10.3390/cimb47030177)
Supplement: Supplementary file 1 [file cimb-47-00177-s001.zip › cimb-3446689-supplementary.pdf]

Supplementary File S1. Identification of the153 studies used to create Table 1 and Figure 2 in this review paper.

1. Raza, M.A.S.; Aslam, M.U.; Valipour, M.; Iqbal, R.; Haider, I.; Mustafa, A.E.-Z.M.A.; Elshikh, M.S.; Ali, I.; Roy, R.; Elshamly, A.M.S. Seed Priming with Selenium Improves Growth and Yield of Quinoa Plants Suffering Drought. *Sci Rep* **2024**, *14*, 886, doi:10.1038/s41598-024-51371-6.
2. Zulfiqar, F.; Moosa, A.; Ferrante, A.; Darras, A.; Ahmed, T.; Jalil, S.; Al-Ashkar, I.; Sabagh, A.E. Melatonin Seed Priming Improves Growth and Physio-Biochemical Aspects of Zinnia Elegans under Salt Stress. *Scientia Horticulturae* **2024**, *323*, 112495, doi:10.1016/j.scienta.2023.112495.
3. Muhie, S.H.; Akele, F.; Yeshiwas, T. Economic Feasibility of Carrot (Daucus Carota L) Production in Response to Different Seed Priming Techniques under Deficit Irrigation. *Scientia Horticulturae* **2024**, *325*, 112662, doi:10.1016/j.scienta.2023.112662.
4. García-Locascio, E.; Valenzuela, E.I.; Cervantes-Avilés, P. Impact of Seed Priming with Selenium Nanoparticles on Germination and Seedlings Growth of Tomato. *Sci Rep* **2024**, *14*, 6726, doi:10.1038/s41598-024-57049-3.
5. Nawaz, H.; Rehman, H.; Ihsan, M.Z.; Rizwan, M.S.; Hussain, N.; Ali, B.; Iqbal, R.; Hasnain, M.U.; Elshikh, M.S.; Alkahtani, J.; et al. Organic Seed Priming with Curtailed Seed Rate Compensated Wheat Grains Productivity by Upgrading Anti-Oxidant Status against Terminal Drought at Flowering and Milking. *Sci Rep* **2024**, *14*, 4941, doi:10.1038/s41598-024-54767-6.
6. de Camargo Santos, A.; Schaffer, B.; Ioannou, A.G.; Moon, P.; Shahid, M.; Rowland, D.; Tillman, B.; Bremgartner, M.; Fotopoulos, V.; Bassil, E. Melatonin Seed Priming Improves Early Establishment and Water Stress Tolerance of Peanut. *Plant Physiology and Biochemistry* **2024**, *211*, 108664, doi:10.1016/j.plaphy.2024.108664.
7. Sharma, V.; Kharb, V.; Verma, V.; Dhaliwal, S.S.; Kalia, A.; Behera, S.K.; Singh, P. Comparative Potential of Different Fe Sources for Seed Priming to Enhance Yield and Iron Content in Direct Seeded Aerobic Rice. *CEREAL RESEARCH COMMUNICATIONS* **2024**, doi:10.1007/s42976-024-00503-9.
8. Adhikari, B.; Olorunwa, O.J.; Horgan, T.E.; Wilson, J.; Barickman, T.C.; Li, T.; Bheemanahalli, R. Seed Priming Attenuates the Impact of Salt Stress and Enhances Lettuce Yields. *Journal of Agriculture and Food Research* **2024**, *15*, 100947, doi:10.1016/j.jafr.2023.100947.
9. Kim, D.-Y.; Kim, M.; Sung, J.-S.; Koduru, J.R.; Nile, S.H.; Syed, A.; Bahkali, A.H.; Seth, C.S.; Ghodake, G.S. Extracellular Synthesis of Silver Nanoparticle Using Yeast Extracts: Antibacterial and Seed Priming Applications. *Appl Microbiol Biotechnol* **2024**, *108*, 150, doi:10.1007/s00253-023-12920-7.
10. De Los Ángeles Sariñana-Navarrete, M.; Benavides-Mendoza, A.; González-Morales, S.; Juárez-Maldonado, A.; Preciado-Rangel, P.; Sánchez-Chávez, E.; Cadenas-Pliego, G.; Antonio-Bautista, A.; Morelos-Moreno, Á. Selenium Seed Priming and Biostimulation Influence the Seed Germination and Seedling Morphology of Jalapeño (Capsicum Annuum L.). *Horticulturae* **2024**, *10*, 119, doi:10.3390/horticulturae10020119.
11. Karalija, E.; Lošić, A.; Demir, A.; Šamec, D. Effects of Seed Priming on Mitigating the Negative Effects of Increased Salinity in Two Varieties of Sweet Pepper (Capsicum Annuum L.). *Soil Systems* **2024**, *8*, doi:10.3390/soilsystems8010035.
12. Zhang, Y.; He, Z.; Xing, P.; Luo, H.; Yan, Z.; Tang, X. Effects of Paclobutrazol Seed Priming on Seedling Quality, Photosynthesis, and Physiological Characteristics of Fragrant Rice. *BMC Plant Biology* **2024**, *24*, 53, doi:10.1186/s12870-023-04683-0.
13. Fregonezi, B.F.; Pereira, A.E.S.; Ferreira, J.M.; Fraceto, L.F.; Gomes, D.G.; Oliveira, H.C. Seed Priming with Nanoencapsulated Gibberellic Acid Triggers Beneficial Morphophysiological and

Biochemical Responses of Tomato Plants under Different Water Conditions. *Agronomy* **2024**, *14*, 588, doi:10.3390/agronomy14030588.
14. Heidarieh, Z.; Jafari, A.; Ebrahimi, H.R.; Jafari Haghighi, B.; Miri, H.R. Seed Priming Alleviates the Adverse Effects of Drought Stress on Sesame Genotypes by Improving Biochemical and Physiological Characteristics. *South African Journal of Botany* **2024**, *167*, 256–269, doi:10.1016/j.sajb.2024.02.023.
15. Mawale, K.S.; Nandini, B.; Giridhar, P. Copper and Silver Nanoparticle Seed Priming and Foliar Spray Modulate Plant Growth and Thrips Infestation in *Capsicum* Spp. *ACS Omega* **2024**, acsomega.3c06961, doi:10.1021/acsomega.3c06961.
16. Ben Youssef, R.; Jelali, N.; Martínez-Andújar, C.; Abdelly, C.; Hernández, J.A. Salicylic Acid and Calcium Chloride Seed Priming: A Prominent Frontier in Inducing Mineral Nutrition Balance and Antioxidant System Capacity to Enhance the Tolerance of Barley Plants to Salinity. *Plants* **2024**, *13*, 1268, doi:10.3390/plants13091268.
17. Hussain, S.; Nisar, F.; Gul, B.; Hameed, A. Seed Priming with Melatonin Improved Salinity Tolerance of Halophytes during Early Life-Cycle Stages. *Plant Growth Regul* **2024**, doi:10.1007/s10725-023-01110-0.
18. Kaya, M.D.; Ergin, N.; Harmancı, P.; Kulan, E.G. Seed Priming as a Method of Preservation and Restoration of Sunflower Seeds. *OCL* **2024**, *31*, 4, doi:10.1051/ocl/2024003.
19. Kumar, R.; Dadhich, A.; Dhiman, M.; Sharma, L.; Sharma, M.M. Stimulatory Effect of ZnO Nanoparticles as a Nanofertilizer in Seed Priming of Pearl Millet (Pennisetum Glaucum) and Their Bioactivity Studies. *South African Journal of Botany* **2024**, *165*, 30–38, doi:10.1016/j.sajb.2023.12.001.
20. Alshegaihi, R.M.; Mfarrej, M.F.B.; Alatawi, A.; Alwutayd, K.M.; Albalawi, S.; Saleem, S.; Saleem, M.H.; Sarfraz, W.; Ali, S.; Abeed, A.H.A. Seed Priming with Iron Oxide Nanoparticles Ameliorates As Toxicity by Decreasing Organic Acid Exudation Pattern and Modulating Specific Gene Expression in Rapeseed (Brassica Napus L.). *Journal of Plant Growth Regulation* **2024**, doi:10.1007/s00344-024-11345-4.
21. Rehman, Z.U.; Haq, R.U.; Ullah, S.; Iqbal, A.; Khan, A.M.; Assogba, C.M.A.; Awais, M. Enhancing Growth and Yield Parameters of *Sinapis Alba* through Optimized Seed Priming Techniques. *Agrosystems Geosci & Env* **2024**, *7*, e20466, doi:10.1002/agg2.20466.
22. Habibi, N.; Terada, N.; Sanada, A.; Koshio, K. Alleviating Salt Stress in Tomatoes through Seed Priming with Polyethylene Glycol and Sodium Chloride Combination. *Stresses* **2024**, *4*, 210–224, doi:10.3390/stresses4020012.
23. Waqas Mazhar, M.; Ishtiaq, M.; Maqbool, M.; Mahmoud, E.A.; Ullah, F.; Elansary, H.O. Optimizing Bitter Gourd (Momordica Charantia L.) Performance: Exploring the Impact of Varied Seed Priming Durations and Zinc Oxide Nanoparticle Concentrations on Germination, Growth, Phytochemical Attributes, and Agronomic Outcomes. *Cogent Food & Agriculture* **2024**, *10*, 2313052, doi:10.1080/23311932.2024.2313052.
24. Zhang, K.; Khan, M.N.; Luo, T.; Bi, J.; Hu, L.; Luo, L. Seed Priming with Gibberellic Acid and Ethephon Improved Rice Germination under Drought Stress via Reducing Oxidative and Cellular Damage. *J Soil Sci Plant Nutr* **2024**, doi:10.1007/s42729-024-01691-3.
25. Fu, Y.; Li, P.; Si, Z.; Ma, S.; Gao, Y. Seeds Priming with Melatonin Improves Root Hydraulic Conductivity of Wheat Varieties under Drought, Salinity, and Combined Stress. *IJMS* **2024**, *25*, 5055, doi:10.3390/ijms25095055.
26. Vanitha, C.; Kathiravan, M.; Umarani, R.; Sathiya, K.; Menaka, C.; Yuvaraj, M.; Cyriac, J. Seed Priming with Nano Silica Alleviates Drought Stress through Regulating Antioxidant Defense System and Osmotic Adjustment in Soybean (Glycine Max L.). *Silicon* **2024**, *16*, 2157–2170, doi:10.1007/s12633-023-02826-4.

27. Krishnasamy, R.; Natesh, R.; Obbineni, J.M. Efficient ROS Scavenging Improves the Growth and Yield in Black Gram (Vigna Mungo (L.) Hepper) after Seed Priming and Treatment Using Biosynthesized Silver Nanoparticles with Pongamia Pinnata (L.) Pierre Leaf Extract. *J Plant Growth Regul* **2024**, doi:10.1007/s00344-024-11276-0.
28. Adeel, M.A.; Hussain, S.; Basit, A.; Hussain, M.B.; Aon, M. Biofortification of Wheat in Salt-Affected Soil through Seed Priming and Soil Application of Zinc. *Journal of Trace Elements and Minerals* **2024**, *8*, 100159, doi:10.1016/j.jtemin.2024.100159.
29. Abdulbaki, A.S.; Alsamadany, H.; Alzahrani, Y.; F Alharby, H.; Olayinka, B.U. Seed Priming of Pepper (Capsicum Annuum L.) with β-Aminobutyric Acid (BABA) Alleviates Drought Stress. *PAK. J. BOT.* **2024**, *56*, doi:10.30848/PJB2024-2(31).
30. Zafar, S.; Khan, S.; Ibrar, D.; Khan, M.K.; Hasnain, Z.; Mehmood, K.; Rais, A.; Gul, S.; Irshad, S.; Nawaz, M. Application of Zinc Nanoparticles as Seed Priming Agent Improves Growth and Yield of Wheat Seedlings Grown under Salinity Stress by Enhanced Antioxidants Activities and Gas Exchange Attributes. *CEREAL RESEARCH COMMUNICATIONS* **2024**, doi:10.1007/s42976-024-00499-2.
31. Silva, B.N.P.; Masetto, T.E.; Rocha, L.G. An Insight into Seed Priming Response of Crotalaria Ochroleuca and Crotalaria Spectabilis during Storage. *Braz. J. Biol.* **2024**, *84*, e279806, doi:10.1590/1519-6984.279806.
32. Usman, M.; Khalid, M.U.; Hasnain, M.; Tauseef, M.; Raza, A.; Akram, M.; Shahid, M.; Ahmad, A.; Ismail, M.S.; Afzal, R.; et al. Effect of Seed Priming on Production of Wheat under Different Tillage Operations. *SJA* **2024**, *40*, doi:10.17582/journal.sja/2024/40.1.133.141.
33. Gaonkar, S.S.; Sincinelli, F.; Balestrazzi, A.; Pagano, A. Quercetin and Rutin as Tools to Enhance Antioxidant Profiles and Post-Priming Seed Storability in Medicago Truncatula. *Agriculture* **2024**, *14*, 738, doi:10.3390/agriculture14050738.
34. Hussain, S.; Ahmed, S.; Akram, W.; Sardar, R.; Abbas, M.; Yasin, N.A. Selenium-Priming Mediated Growth and Yield Improvement of Turnip under Saline Conditions. *International Journal of Phytoremediation* **2024**, *26*, 710–726, doi:https://doi.org/10.1080/15226514.2023.2261548.
35. Wang, R.; Li, C.; Zeng, L.; Liu, L.; Xi, J.; Li, J. Polyethylene Glycol Priming Enhances the Seed Germination and Seedling Growth of Scutellaria Baicalensis Georgi under Salt Stress. *Plants* **2024**, *13*, 565, doi:10.3390/plants13050565.
36. Haque, Md.A. Effect of Seed Priming on Germination Behavior and Emergence of Wheat (Triticum Aestivum L.). *JAERI* **2024**, *25*, 53–61, doi:10.9734/jaeri/2024/v25i2584.
37. Sidhu, A.S.; Chhina, G.S.; Bhullar, R.K. Impact of Different Methods of Sowing and Seed Priming on Yield Attributed Characters of Rice (Oryza Sativa L.). *AJSSPN* **2024**, *10*, 105–109, doi:10.9734/ajsspn/2024/v10i2266.
38. Shadmehri, A.; Abbasdokht, H. Investigating Seed Priming on Some Physiological Characteristics and Yield of Three Genotypes (Q12, Q29 and Giza1) of Quinoa (*Chenopodium Quinoa* Willd) under Different Irrigation Regimes. [illegible] **2024**, doi:10.22077/escs.2023.5995.2185.
39. Wang, J.; Song, J.; Wu, X.; Deng, Q.; Zhu, Z.; Ren, M.; Ye, M.; Zeng, R. Wheat (Triticum Aestivum L.) Seed Priming with Calcium Chloride Enhances Resistance to Wheat Aphid (Schizaphis Graminum Rondani, Hemiptera: Aphididae) 2024.
40. Firozabad, B.S.; Ghiyasi, M.; Amirnia, R. The Effect of Seed Priming and Foliar Application in Conventional and Nano Forms on Quantitative and Qualitative Performance of Safflower. *Journal of Crops Improvement* **2024**, *26*, 109–124, doi:https://doi.org/10.22059/jci.2023.358846.2827.

41. Jacob, M.E.; Nair, D.; Sreekala, G.S.; Anith, K.N.; Alex, S.; Rani, T.S.; Viji, M.M. Chitosan Seed Priming Enhanced Germination and Seedling Growth in Ashwagandha (*Withania Somnifera* (L.) Dunal.). *Internation Journal of Phytomedicines and Related Industries* **2024**, *16*, 119–127, doi:http://dx.doi.org/10.5958/0975-6892.2024.00013.3.
42. Amirikia, F.; Nabipour, M.; Farzaneh, M. Effect of Seed Priming on Germination, Total Dry Matter and Some Physiological Traits in Two Alhagi Species (Alhagi Maurorum Esfahan Ecotype and A. Graecorum Khuzestan Ecotype) under Saline Conditions. *Environmental Stresses in Crop Sciences* **2024**, doi:10.22077/escs.2023.5884.2181.
43. Bano, N.; Khan, S.; Hamid, Y.; Bano, F.; Khan, A.G.; Asmat Ullah, M.; Li, T.; Ullah, H.; Bolan, N.; Rinklebe, J.; et al. Seed Nano-Priming with Multiple Nanoparticles Enhanced the Growth Parameters of Lettuce and Mitigated Cadmium (Cd) Bio-Toxicity: An Advanced Technique for Remediation of Cd Contaminated Environments. *Environmental Pollution* **2024**, *344*, 123300, doi:10.1016/j.envpol.2024.123300.
44. Kaushal, K.; Rajani, K.; Kumar, R.R.; Ranjan, T.; Kumar, A.; Ahmad, M.F.; Kumar, V.; Kumar, V.; Kumar, A. Physio-Biochemical Responses and Crop Performance Analysis in Chickpea upon Botanical Priming. *Sci Rep* **2024**, *14*, 9342, doi:10.1038/s41598-024-59878-8.
45. Afifa, R.I.; Islam, N.; Choudhury, S. Effects of Priming on Onion Seed Germination and Field Performance during Summer Sowing. *JSRR* **2024**, *30*, 252–258, doi:10.9734/jsrr/2024/v30i41911.
46. Zhang, H.; Zhang, X.; Gao, G.; Ali, I.; Wu, X.; Tang, M.; Chen, L.; Jiang, L.; Liang, T. Effects of Various Seed Priming on Morphological, Physiological, and Biochemical Traits of Rice under Chilling Stress. *Front. Plant Sci.* **2023**, *14*, 1146285, doi:10.3389/fpls.2023.1146285.
47. Alhammad, B.A.; Ahmad, A.; Seleiman, M.F.; Tola, E. Seed Priming with Nanoparticles and 24-Epibrassinolide Improved Seed Germination and Enzymatic Performance of Zea Mays L. in Salt-Stressed Soil. *Plants* **2023**, *12*, 690, doi:10.3390/plants12040690.
48. Kakar, H.A.; Ullah, S.; Shah, W.; Ali, B.; Satti, S.Z.; Ullah, R.; Muhammad, Z.; Eldin, S.M.; Ali, I.; Alwahibi, M.S.; et al. Seed Priming Modulates Physiological and Agronomic Attributes of Maize (*Zea Mays* L.) under Induced Polyethylene Glycol Osmotic Stress. *ACS Omega* **2023**, *8*, 22788–22808, doi:10.1021/acsomega.3c01715.
49. Tamindžić, G.; Ignjatov, M.; Miljaković, D.; Červenski, J.; Milošević, D.; Nikolić, Z.; Vasiljević, S. Seed Priming Treatments to Improve Heat Stress Tolerance of Garden Pea (Pisum Sativum L.). *Agriculture* **2023**, *13*, 439, doi:10.3390/agriculture13020439.
50. Sheferie, M. Effect of Seed Priming Methods on Seed Quality of Okra (Abelmoschus Esculentus (L.) Moench) Genotypes. *Advances in Agriculture* **2023**, *2023*, 1–9, doi:10.1155/2023/3951752.
51. Soni, A.T.; Rookes, J.E.; Arya, S.S. Chitosan Nanoparticles as Seed Priming Agents to Alleviate Salinity Stress in Rice (Oryza Sativa L.) Seedlings. *Polysaccharides* **2023**, *4*, 129–141, doi:10.3390/polysaccharides4020010.
52. Khalequzzaman; Ullah, H.; Himanshu, S.K.; Islam, N.-E.-T.; Tisarum, R.; Cha-um, S.; Datta, A. Seed Priming Improves Germination, Yield, and Water Productivity of Cotton Under Drought Stress. *J Soil Sci Plant Nutr* **2023**, *23*, 2418–2432, doi:10.1007/s42729-023-01196-5.
53. Monajjem, S.; Soltani, E.; Zainali, E.; Esfahani, M.; Ghaderi-Far, F.; Chaleshtori, M.H.; Rezaei, A. Seed Priming Improves Enzymatic and Biochemical Performances of Rice During Seed Germination under Low and High Temperatures. *Rice Science* **2023**, *30*, 335–347, doi:10.1016/j.rsci.2023.03.012.
54. Hussain, S.; Ahmed, S.; Akram, W.; Li, G.; Yasin, N.A. Selenium Seed Priming Enhanced the Growth of Salt-Stressed Brassica Rapa L. through Improving Plant Nutrition and the Antioxidant System. *Front. Plant Sci.* **2023**, *13*, 1050359, doi:10.3389/fpls.2022.1050359.

55. Cappetta, E.; Del Regno, C.; Conte, M.; Castro-Hinojosa, C.; Del Sol-Fernández, S.; Vergata, C.; Buti, M.; Curcio, R.; Onder, A.; Mazzei, P.; et al. An Integrated Multilevel Approach Unveils Complex Seed–Nanoparticle Interactions and Their Implications for Seed Priming. *ACS Nano* **2023**, *17*, 22539–22552, doi:10.1021/acsnano.3c06172.
56. Xia, J.; Hao, X.; Wang, T.; Li, H.; Shi, X.; Liu, Y.; Luo, H. Seed Priming with Gibberellin Regulates the Germination of Cotton Seeds Under Low-Temperature Conditions. *J Plant Growth Regul* **2023**, *42*, 319–334, doi:10.1007/s00344-021-10549-2.
57. Doddagoudar, S.R.; Nagaraja, M.; Lakshmikanth, M.; Srininvas, A.G.; Shakuntala, N.M.; Umesh, H.; Mahanthshivayogayya, M. Improving the Resilience of Rice Seedlings to Low Temperature Stress through Seed Priming. *South African Journal of Botany* **2023**, *162*, 183–192, doi:10.1016/j.sajb.2023.09.005.
58. Faraz, A.; Faizan, M.; D. Rajput, V.; Minkina, T.; Hayat, S.; Faisal, M.; Alatar, A.A.; Abdel-Salam, E.M. CuO Nanoparticle-Mediated Seed Priming Improves Physio-Biochemical and Enzymatic Activities of Brassica Juncea. *Plants* **2023**, *12*, 803, doi:10.3390/plants12040803.
59. Abdelkader, M.; Voronina, L.; Puchkov, M.; Shcherbakova, N.; Pakina, E.; Zargar, M.; Lyashko, M. Seed Priming with Exogenous Amino Acids Improves Germination Rates and Enhances Photosynthetic Pigments of Onion Seedlings (Allium Cepa L.). *Horticulturae* **2023**, *9*, 80, doi:10.3390/horticulturae9010080.
60. Ishtiaq, M.; Mazhar, M.W.; Maqbool, M.; Hussain, T.; Hussain, S.A.; Casini, R.; Abd-ElGawad, A.M.; Elansary, H.O. Seed Priming with the Selenium Nanoparticles Maintains the Redox Status in the Water Stressed Tomato Plants by Modulating the Antioxidant Defense Enzymes. *Plants* **2023**, *12*, 1556, doi:10.3390/plants12071556.
61. Lyalina, T.; Shagdarova, B.; Zhuikova, Y.; Il'ina, A.; Lunkov, A.; Varlamov, V. Effect of Seed Priming with Chitosan Hydrolysate on Lettuce (Lactuca Sativa) Growth Parameters. *Molecules* **2023**, *28*, 1915, doi:10.3390/molecules28041915.
62. El-Hawary, M.M.; Hashem, O.S.M.; Hasanuzzaman, M. Seed Priming and Foliar Application with Ascorbic Acid and Salicylic Acid Mitigate Salt Stress in Wheat. *Agronomy* **2023**, *13*, 493, doi:10.3390/agronomy13020493.
63. Soares, T.F.S.N.; Muniz, E.N.; Sousa, J.P.S.; Oliveira Júnior, L.F.G. de; Barbosa, A.M.; Silva, A.V.C. da Seed Priming as a Strategy to Increase the Performance of Drumstick Tree. *South African Journal of Botany* **2023**, *157*, 279–286, doi:10.1016/j.sajb.2023.03.037.
64. Gour, T.; Sharma, A.; Lal, R.; Heikrujam, M.; Gupta, A.; Agarwal, L.K.; Chetri, S.P.K.; Kumar, R.; Sharma, K. Amelioration of the Physio-Biochemical Responses to Salinity Stress and Computing the Primary Germination Index Components in Cauliflower on Seed Priming. *Heliyon* **2023**, *9*, e14403, doi:10.1016/j.heliyon.2023.e14403.
65. Ellouzi, H.; Zorrig, W.; Amraoui, S.; Oueslati, S.; Abdelly, C.; Rabhi, M.; Siddique, K.H.M.; Hessini, K. Seed Priming with Salicylic Acid Alleviates Salt Stress Toxicity in Barley by Suppressing ROS Accumulation and Improving Antioxidant Defense Systems, Compared to Halo- and Gibberellin Priming. *Antioxidants* **2023**, *12*, 1779, doi:10.3390/antiox12091779.
66. Alam, A.U.; Ullah, H.; Himanshu, S.K.; Tisarum, R.; Cha-um, S.; Datta, A. Seed Priming Enhances Germination and Morphological, Physio-Biochemical, and Yield Traits of Cucumber under Water-Deficit Stress. *J Soil Sci Plant Nutr* **2023**, *23*, 3961–3978, doi:10.1007/s42729-023-01314-3.
67. Chen, S.; Liu, H.; Yangzong, Z.; Gardea-Torresdey, J.L.; White, J.C.; Zhao, L. Seed Priming with Reactive Oxygen Species-Generating Nanoparticles Enhanced Maize Tolerance to Multiple Abiotic Stresses. *Environ. Sci. Technol.* **2023**, *57*, 19932–19941, doi:10.1021/acs.est.3c07339.

68. Ahmad, A.; Tola, E.; Alshahrani, T.S.; Seleiman, M.F. Enhancement of Morphological and Physiological Performance of Zea Mays L. under Saline Stress Using ZnO Nanoparticles and 24-Epibrassinolide Seed Priming. *Agronomy* **2023**, *13*, 771, doi:10.3390/agronomy13030771.
69. Mazhar, M.W.; Ishtiaq, M.; Maqbool, M.; Akram, R. Seed Priming with Zinc Oxide Nanoparticles Improves Growth, Osmolyte Accumulation, Antioxidant Defence and Yield Quality of Water-Stressed Mung Bean Plants. *Arid Land Research and Management* **2023**, *37*, 222–246, doi:10.1080/15324982.2022.2132547.
70. Fathi, N.; Kazemeini, S.A.; Alinia, M.; Mastinu, A. The Effect of Seed Priming with Melatonin on Improving the Tolerance of Zea Mays L. Var Saccharata to Paraquat-Induced Oxidative Stress through Photosynthetic Systems and Enzymatic Antioxidant Activities. *Physiological and Molecular Plant Pathology* **2023**, *124*, 101967, doi:10.1016/j.pmpp.2023.101967.
71. Li, Z.; Liu, Y. Seed Priming Stimulates Germination and Early Seedling Establishment Of *Corethrodendron Multijugum* Under Drought Stress. *Seed Science and Technology* **2023**, *51*, 51–63, doi:10.15258/sst.2023.51.1.06.
72. Hidangmayum, A.; Dwivedi, P.; Kumar, P.; Upadhyay, S.K. Seed Priming and Foliar Application of Chitosan Ameliorate Drought Stress Responses in Mungbean Genotypes Through Modulation of Morpho-Physiological Attributes and Increased Antioxidative Defense Mechanism. *J Plant Growth Regul* **2023**, *42*, 6137–6154, doi:10.1007/s00344-022-10792-1.
73. Youssef, R.B.; Boukari, N.; Abdelly, C.; Jelali, N. Mitigation of Salt Stress and Stimulation of Growth by Salicylic Acid and Calcium Chloride Seed Priming in Two Barley Species. *Plant Biosystems - An International Journal Dealing with all Aspects of Plant Biology* **2023**, *157*, 758–768, doi:10.1080/11263504.2023.2200792.
74. Oğuz, M.Ç.; Oğuz, E.; Güler, M. Seed Priming with Essential Oils for Sustainable Wheat Agriculture in Semi-Arid Region. *PeerJ* **2023**, *11*, e15126, doi:10.7717/peerj.15126.
75. Salehi, H.; Cheheregani Rad, A.; Raza, A.; Djalovic, I.; Prasad, P.V.V. The Comparative Effects of Manganese Nanoparticles and Their Counterparts (Bulk and Ionic) in Artemisia Annua Plants via Seed Priming and Foliar Application. *Front. Plant Sci.* **2023**, *13*, 1098772, doi:10.3389/fpls.2022.1098772.
76. Ul Haq, T.; Ullah, R.; Khan, M.N.; Nazish, M.; Almutairi, S.M.; Rasheed, R.A. Seed Priming with Glutamic-Acid-Functionalized Iron Nanoparticles Modulating Response of Vigna Radiata (L.) R. Wilczek (Mung Bean) to Induce Osmotic Stress. *Micromachines* **2023**, *14*, 736, doi:10.3390/mi14040736.
77. Hidangmayum, A.; Dwivedi, P. Effect of Chitosan Seed Priming on Mungbean Seedlings Subjected to Different Levels of Water Potential. *Acta Physiol Plant* **2023**, *45*, 6, doi:10.1007/s11738-022-03483-7.
78. Dawoud, T.M.; Akhtar, N.; Okla, M.K.; Shah, A.N.; Shah, A.A.; Abdel-Mawgoud, M.; AbdElgayed, G.; Al-Hashimi, A.; AbdElgawad, H. Seed Priming with Pomegranate Peel Extract Improves Growth, Glucosinolates Metabolism and Antimicrobial Potential of Brassica Oleraceae Varieties. *J Plant Growth Regul* **2023**, *42*, 3043–3055, doi:10.1007/s00344-022-10769-0.
79. Mazhar, M.W.; Ishtiaq, M.; Maqbool, M.; Ullah, F.; Sayed, S.R.M.; Mahmoud, E.A. Seed Priming with Iron Oxide Nanoparticles Improves Yield and Antioxidant Status of Garden Pea (Pisum Sativum L.) Grown under Drought Stress. *South African Journal of Botany* **2023**, *162*, 577–587, doi:10.1016/j.sajb.2023.09.047.
80. Shumaila; Ullah, S.; Shah, W.; Hafeez, A.; Ali, B.; Khan, S.; Ercisli, S.; Al-Ghamdi, A.A.; Elshikh, M.S. Biochar and Seed Priming Technique with Gallic Acid: An Approach toward Improving Morpho-Anatomical and Physiological Features of *Solanum Melongena* L. under Induced NaCl and Boron Stresses. *ACS Omega* **2023**, *8*, 28207–28232, doi:10.1021/acsomega.3c01720.

81. Hadia, E.; Slama, A.; Romdhane, L.; Cheikh M'Hamed, H.; Fahej, M.A.S.; Radhouane, L. Seed Priming of Bread Wheat Varieties with Growth Regulators and Nutrients Improves Salt Stress Tolerance Particularly for the Local Genotype. *J Plant Growth Regul* **2023**, *42*, 304–318, doi:10.1007/s00344-021-10548-3.
82. Al Hinai, M.S.; Ullah, A.; Al-Toubi, A.-K.M.; Al Harrasi, I.R.; Alamri, A.A.; Farooq, M. Co-Application of Biochar and Seed Priming with Nano-Sized Chitosan-Proline Improves Salt Tolerance in Differentially Responding Bread Wheat Genotypes. *J Soil Sci Plant Nutr* **2023**, *23*, 3058–3073, doi:10.1007/s42729-023-01276-6.
83. Mulaudzi, T.; Sias, G.; Nkuna, M.; Ndou, N.; Hendricks, K.; Ikebudu, V.; Koo, A.J.; Ajayi, R.F.; Iwuoha, E. Seed Priming with MeJa Prevents Salt-Induced Growth Inhibition and Oxidative Damage in Sorghum Bicolor by Inducing the Expression of Jasmonic Acid Biosynthesis Genes. *IJMS* **2023**, *24*, 10368, doi:10.3390/ijms241210368.
84. Ahmed, S.; Amjad, M.; Sardar, R.; Siddiqui, M.H.; Irfan, M. Seed Priming with Triacontanol Alleviates Lead Stress in Phaseolus Vulgaris L. (Common Bean) through Improving Nutritional Orchestration and Morpho-Physiological Characteristics. *Plants* **2023**, *12*, 1672, doi:10.3390/plants12081672.
85. Ran, M.; Lu, Y.; Wu, J.; Li, J. Mitigating Antimony Toxicity in Rice (Oryza Sativa L.) Through Exogenous Selenium Supplementation: A Comparative Study of Seed Priming, Hydroponics, and Foliar Spray Methods. *J Plant Growth Regul* **2024**, *43*, 816–828, doi:10.1007/s00344-023-11141-6.
86. Sghayar, S.; Debez, A.; Lucchini, G.; Abruzzese, A.; Zorrig, W.; Negrini, N.; Morgutti, S.; Abdelly, C.; Sacchi, G.A.; Pecchioni, N.; et al. Seed Priming Mitigates High Salinity Impact on Germination of Bread Wheat (*Triticum aestivum* L.) by Improving Carbohydrate and Protein Mobilization. *Plant Direct* **2023**, *7*, e497, doi:10.1002/pld3.497.
87. Rachmawati, D.; Aisy, S.P.; Novanursandy, N.B. Effect of Seed Priming on Growth and Physiological Responses of Chili Pepper (Capsicum Frutescens L.) under Salinity Stress. *IOP Conf. Ser.: Earth Environ. Sci.* **2023**, *1165*, 012016, doi:10.1088/1755-1315/1165/1/012016.
88. Tyagi, K.; V, P.; Tyagi, P.; Kumari, A.; Pandey, R.; Meena, N.L.; Khan, M.I.R.; Tyagi, A.; Maheshwari, C. Seed Priming with Melatonin Induces Rhizogenesis and Modulates Physio-Biochemical Traits in High-Yielding Rice (Oryza Sativa L.) Genotypes. *South African Journal of Botany* **2023**, *163*, 191–200, doi:10.1016/j.sajb.2023.10.043.
89. Ashraf Ganjouii, F.; Nasibi, F.; Manoochehri Kalantari, K.; Ahmadi Mousavi, E. Effect of Seed Priming with Selenium Nanoparticles and Plant Growth Promoting Rhizobacteria on Improving Quinoa Seedling Growth under Salinity Stress. *jispp* **2023**, *11*, 65–74.
90. Nie, M.; Ning, N.; Liang, D.; Zhang, H.; Li, S.; Li, S.; Fan, X.; Zhang, Y. Seed Priming with Selenite Enhances Germination and Seedling Growth of Sorghum [*Sorghum Bicolor* (L.) Moench] under Salt Stress. *Acta Agriculturae Scandinavica, Section B — Soil & Plant Science* **2023**, *73*, 42–53, doi:10.1080/09064710.2023.2177561.
91. Habibi, N.; Aryan, S.; Amin, M.W.; Sanada, A.; Terada, N.; Koshio, K. Potential Benefits of Seed Priming under Salt Stress Conditions on Physiological, and Biochemical Attributes of Micro-Tom Tomato Plants. *Plants* **2023**, *12*, 2187, doi:10.3390/plants12112187.
92. Charachimwe, R.R.; Chandiposha, M.; Manjeru, P. The Effect of Seed Priming to Improve Germination Parameters and Early Growth of Chickpea (Cicer Arietnum L). *International Journal of Agronomy* **2023**, *2023*, 1–8, doi:10.1155/2023/1178679.
93. Moradi, L.; Siosemardeh, A. Combination of Seed Priming and Nutrient Foliar Application Improved Physiological Attributes, Grain Yield, and Biofortification of Rainfed Wheat. *Front. Plant Sci.* **2023**, *14*, 1287677, doi:10.3389/fpls.2023.1287677.

94. Kharb, V.; Sharma, V.; Dhaliwal, S.S.; Kalia, A. Influence of Iron Seed Priming on Seed Germination, Growth and Iron Content in Rice Seedlings. *Journal of Plant Nutrition* **2023**, *46*, 4054–4062, doi:10.1080/01904167.2023.2220731.
95. Selim, S.; Zrig, A.; Albqmi, M.; M. Al-Sanea, M.; Alnusaire, T.S.; Almuhayawi, M.S.; Jaouni, S.K.A.; Hussein, S.; Warrad, M.; AbdElgawad, H. BAP (6-Benzylaminopurine) Seed-Priming Enhanced Growth, Antioxidant Accumulation and Anthocyanin Metabolism in Olive Sprouts. *Horticulturae* **2023**, *9*, 1055, doi:10.3390/horticulturae9091055.
96. Hussain, S.; Ahmed, S.; Yasin, N.A.; Akram, W.; Sardar, R.; Ahmad, A.; Li, G. In Vitro and in Silico Study of Salt Stress Resilience in Brassica Rapa through Selenium Seed Priming. *South African Journal of Botany* **2023**, *160*, 504–515, doi:10.1016/j.sajb.2023.07.024.
97. Alam, A.U.; Ullah, H.; Himanshu, S.K.; Praseartkul, P.; Tisarum, R.; Cha-um, S.; Datta, A. Seed Priming and Foliar Application of Salicylic Acid Is Equally Beneficial in Mitigating Drought Stress in Cucumber. *J Soil Sci Plant Nutr* **2023**, *23*, 6299–6316, doi:10.1007/s42729-023-01485-z.
98. Ashraf, M.A.; Ibrahim, S.M.; Rasheed, R.; Rizwan, M.; Hussain, I.; Ali, S. Effect of Seed Priming by Taurine on Growth and Chromium (Cr) Uptake in Canola (Brassica Napus L.) under Cr Stress. *Environ Sci Pollut Res* **2023**, *30*, 87851–87865, doi:10.1007/s11356-023-28471-3.
99. Ceritoglu, M.; Erman, M.; Çığ, F.; Ceritoglu, F.; Uçar, Ö.; Soysal, S.; El Sabagh, A. Enhancement of Root System Architecture,Seedling Growth, and Germination in Lentilunder Salinity Stress by Seed Priming with Siliconand Salicylic Acid. *Pol. J. Environ. Stud.* **2023**, *32*, 4481–4491, doi:10.15244/pjoes/168941.
100. Ashraf, M.Y.; Nisa, Z.-U.; Ali, N.; Shani, M.Y.; Naz, A.; Azmat, M.; Ashraf, I. Salicylic Acid Seed Priming Improved Dry Biomass and Ionic Efficiency of Mungbean [Vigna Radiata (L.) Wilczek] under Salt Stress Conditions. *PAK. J. BOT.* **2023**, *55*, doi:10.30848/PJB2023-5(7).
101. Lee, D.-H.; Kim, S.-H.; Tolera, B.; Heo, J.-Y. Effect of Seed Priming on the Germination Properties of Allium Hookeri. *International Journal of Horticultural Science and Technology* **2023**, *10*, 203–210, doi:https://doi.org/10.22059/ijhst.2022.342487.559.
102. MacDonald, M.T.; Kannan, R.; Jayaseelan, R. Ascorbic Acid Preconditioning Effect on Broccoli Seedling Growth and Photosynthesis under Drought Stress. *Plants* **2022**, *11*, 1324, doi:10.3390/plants11101324.
103. Tu, K.; Cheng, Y.; Pan, T.; Wang, J.; Sun, Q. Effects of Seed Priming on Vitality and Preservation of Pepper Seeds. *Agriculture* **2022**, *12*, 603, doi:10.3390/agriculture12050603.
104. Adhikari, B.; Olorunwa, O.J.; Barickman, T.C. Seed Priming Enhances Seed Germination and Morphological Traits of Lactuca Sativa L. under Salt Stress. *Seeds* **2022**, *1*, 74–86, doi:10.3390/seeds1020007.
105. Salam, A.; Khan, A.R.; Liu, L.; Yang, S.; Azhar, W.; Ulhassan, Z.; Zeeshan, M.; Wu, J.; Fan, X.; Gan, Y. Seed Priming with Zinc Oxide Nanoparticles Downplayed Ultrastructural Damage and Improved Photosynthetic Apparatus in Maize under Cobalt Stress. *Journal of Hazardous Materials* **2022**, *423*, 127021, doi:10.1016/j.jhazmat.2021.127021.
106. Zhou, X.; Jia, X.; Zhang, Z.; Chen, K.; Wang, L.; Chen, H.; Yang, Z.; Li, C.; Zhao, L. AgNPs Seed Priming Accelerated Germination Speed and Altered Nutritional Profile of Chinese Cabbage. *Science of The Total Environment* **2022**, *808*, 151896, doi:10.1016/j.scitotenv.2021.151896.
107. Guo, Y.; Li, D.; Liu, L.; Sun, H.; Zhu, L.; Zhang, K.; Zhao, H.; Zhang, Y.; Li, A.; Bai, Z.; et al. Seed Priming With Melatonin Promotes Seed Germination and Seedling Growth of Triticale Hexaploide L. Under PEG-6000 Induced Drought Stress. *Front. Plant Sci.* **2022**, *13*, 932912, doi:10.3389/fpls.2022.932912.
108. Choukri, M.; Abouabdillah, A.; Bouabid, R.; Abd-Elkader, O.H.; Pacioglu, O.; Boufahja, F.; Bourioug, M. Zn Application through Seed Priming Improves Productivity and Grain Nutritional

Quality of Silage Corn. *Saudi Journal of Biological Sciences* **2022**, *29*, 103456, doi:10.1016/j.sjbs.2022.103456.
109. Ramírez, E.; Chaâbene, Z.; Hernández-Apaolaza, L.; Rekik, M.; Elleuch, A.; De La Fuente, V. Seed Priming to Optimize Germination in Arthrocnemum Moq. *BMC Plant Biol* **2022**, *22*, 527, doi:10.1186/s12870-022-03893-2.
110. Ofoe, R.; Gunupuru, L.R.; Wang-Pruski, G.; Fofana, B.; Thomas, R.H.; Abbey, Lord Seed Priming with Pyroligneous Acid Mitigates Aluminum Stress, and Promotes Tomato Seed Germination and Seedling Growth. *Plant Stress* **2022**, *4*, 100083, doi:10.1016/j.stress.2022.100083.
111. Adhikary, S.; Biswas, B.; Chakraborty, D.; Timsina, J.; Pal, S.; Chandra Tarafdar, J.; Banerjee, S.; Hossain, A.; Roy, S. Seed Priming with Selenium and Zinc Nanoparticles Modifies Germination, Growth, and Yield of Direct-Seeded Rice (Oryza Sativa L.). *Sci Rep* **2022**, *12*, 7103, doi:10.1038/s41598-022-11307-4.
112. González-García, Y.; López-Vargas, E.R.; Pérez-Álvarez, M.; Cadenas-Pliego, G.; Benavides-Mendoza, A.; Valdés-Reyna, J.; Pérez-Labrada, F.; Juárez-Maldonado, A. Seed Priming with Carbon Nanomaterials Improves the Bioactive Compounds of Tomato Plants under Saline Stress. *Plants* **2022**, *11*, 1984, doi:10.3390/plants11151984.
113. Tuiwong, P.; Lordkaew, S.; Veeradittakit, J.; Jamjod, S.; Prom-u-thai, C. Seed Priming and Foliar Application with Nitrogen and Zinc Improve Seedling Growth, Yield, and Zinc Accumulation in Rice. *Agriculture* **2022**, *12*, 144, doi:10.3390/agriculture12020144.
114. Wang, Y.; Shen, C.; Jiang, Q.; Wang, Z.; Gao, C.; Wang, W. Seed Priming with Calcium Chloride Enhances Stress Tolerance in Rice Seedlings. *Plant Science* **2022**, *323*, 111381, doi:10.1016/j.plantsci.2022.111381.
115. Khan, M.N.; Li, Y.; Fu, C.; Hu, J.; Chen, L.; Yan, J.; Khan, Z.; Wu, H.; Li, Z. CeO_2 Nanoparticles Seed Priming Increases Salicylic Acid Level and ROS Scavenging Ability to Improve Rapeseed Salt Tolerance. *Global Challenges* **2022**, *6*, 2200025, doi:10.1002/gch2.202200025.
116. Khan, S.; Ibrar, D.; Bashir, S.; Rashid, N.; Hasnain, Z.; Nawaz, M.; Al-Ghamdi, A.A.; Elshikh, M.S.; Dvořáčková, H.; Dvořáček, J. Application of Moringa Leaf Extract as a Seed Priming Agent Enhances Growth and Physiological Attributes of Rice Seedlings Cultivated under Water Deficit Regime. **2022**.
117. Mazhar, M.W.; Ishtiaq, M.; Maqbool, M.; Akram, R. Seed Priming with Calcium Oxide Nanoparticles Improves Germination, Biomass, Antioxidant Defence and Yield Traits of Canola Plants under Drought Stress. *South African Journal of Botany* **2022**, *151*, 889–899, doi:10.1016/j.sajb.2022.11.017.
118. Nie, L.; Song, S.; Yin, Q.; Zhao, T.; Liu, H.; He, A.; Wang, W. Enhancement in Seed Priming-Induced Starch Degradation of Rice Seed Under Chilling Stress via GA-Mediated α-Amylase Expression. *Rice* **2022**, *15*, 19, doi:10.1186/s12284-022-00567-3.
119. Neha; Twinkle; Mohapatra, S.; Sirhindi, G.; Dogra, V. Seed Priming with Brassinolides Improves Growth and Reinforces Antioxidative Defenses under Normal and Heat Stress Conditions in Seedlings of Brassica Juncea. *Physiologia Plantarum* **2022**, *174*, e13814, doi:https://doi.org/10.1111/ppl.13814.
120. Islam, A.T.M.T.; Ullah, H.; Himanshu, S.K.; Tisarum, R.; Cha-um, S.; Datta, A. Effect of Salicylic Acid Seed Priming on Morpho-Physiological Responses and Yield of Baby Corn under Salt Stress. *Scientia Horticulturae* **2022**, *304*, 111304, doi:10.1016/j.scienta.2022.111304.
121. Ray, J.; Bordolui, S.K. Effect of Seed Priming as Pre-Treatment Factors on Germination and Seedling Vigour of Tomato. *IJPSS* **2022**, 302–311, doi:10.9734/ijpss/2022/v34i2031156.

122. Haider, I.; ur Rehman, H. The Impact of Different Seed Priming Agents and Priming Durations on Stand Establishment and Biochemical Attributes of Stevia Rebaudiana Bertoni. *Saudi Journal of Biological Sciences* **2022**, *29*, 2210–2218, doi:10.1016/j.sjbs.2021.11.040.
123. Basit, F.; Ulhassan, Z.; Mou, Q.; Nazir, M.M.; Hu, J.; Hu, W.; Song, W.; Sheteiwy, M.S.; Zhou, W.; Bhat, J.A.; et al. Seed Priming with Nitric Oxide and/or Spermine Mitigate the Chromium Toxicity in Rice (Oryza Sativa) Seedlings by Improving the Carbon-Assimilation and Minimising the Oxidative Damages. *Functional Plant Biol.* **2023**, *50*, 121–135.
124. Ashraf, M.A.; Rasheed, R.; Hussain, I.; Hafeez, A.; Adrees, M.; Rehman, M.Z. ur; Rizwan, M.; Ali, S. Effect of Different Seed Priming Agents on Chromium Accumulation, Oxidative Defense, Glyoxalase System and Mineral Nutrition in Canola (Brassica Napus L.) Cultivars. *Environmental Pollution* **2022**, *309*, 119769, doi:10.1016/j.envpol.2022.119769.
125. Gupta, N.; Singh, P.M.; Sagar, V.; Pandya, A.; Chinnappa, M.; Kumar, R.; Bahadur, A. Seed Priming with ZnO and Fe3O4 Nanoparticles Alleviate the Lead Toxicity in Basella Alba L. through Reduced Lead Uptake and Regulation of ROS. *Plants* **2022**, *11*, 2227, doi:10.3390/plants11172227.
126. Kanjevac, M.; Bojović, B.; Ćirić, A.; Stanković, M.; Jakovljević, D. Seed Priming Improves Biochemical and Physiological Performance of Wheat Seedlings under Low-Temperature Conditions. **2023**.
127. Banerjee, P.; Venugopalan, V.K.; Nath, R.; Chakraborty, P.K.; Gaber, A.; Alsanie, W.F.; Raafat, B.M.; Hossain, A. Seed Priming and Foliar Application of Nutrients Influence the Productivity of Relay Grass Pea (Lathyrus Sativus L.) through Accelerating the Photosynthetically Active Radiation (PAR) Use Efficiency. *Agronomy* **2022**, *12*, 1125, doi:10.3390/agronomy12051125.
128. Das, D.; Basar, N.U.; Ullah, H.; Attia, A.; Salin, K.R.; Datta, A. Growth, Yield and Water Productivity of Rice as Influenced by Seed Priming under Alternate Wetting and Drying Irrigation. *Archives of Agronomy and Soil Science* **2022**, *68*, 1515–1529, doi:10.1080/03650340.2021.1912320.
129. Okello, D.; Komakech, R.; Gang, R.; Rahmat, E.; Chung, Y.; Omujal, F.; Kang, Y. Influence of Various Temperatures, Seed Priming Treatments and Durations on Germination and Growth of the Medicinal Plant Aspilia Africana. *Scientific Reports* **2022**, *12*, 14180, doi:10.1038/s41598-022-18236-2.
130. Farhana; Munis, M.F.H.; Alamer, K.H.; Althobaiti, A.T.; Kamal, A.; Liaquat, F.; Haroon, U.; Ahmed, J.; Chaudhary, H.J.; Attia, H. ZnO Nanoparticle-Mediated Seed Priming Induces Biochemical and Antioxidant Changes in Chickpea to Alleviate Fusarium Wilt. *JoF* **2022**, *8*, 753, doi:10.3390/jof8070753.
131. Khan, I.; Zafar, H.; Chattha, M.U.; Mahmood, A.; Maqbool, R.; Athar, F.; Alahdal, M.A.; Bibi, F.; Mahmood, F.; Hassan, M.U.; et al. Seed Priming with Different Agents Mitigate Alkalinity Induced Oxidative Damage and Improves Maize Growth. *Not Bot Horti Agrobo* **2022**, *50*, 12615, doi:10.15835/nbha50112615.
132. Ling, Y.; Zhao, Y.; Cheng, B.; Tan, M.; Zhang, Y.; Li, Z. Seed Priming with Chitosan Improves Germination Characteristics Associated with Alterations in Antioxidant Defense and Dehydration-Responsive Pathway in White Clover under Water Stress. *Plants* **2022**, *11*, 2015, doi:10.3390/plants11152015.
133. Dhingra, P.; Sharma, S.; Singh, K.H.; Kushwaha, H.S.; Barupal, J.K.; Haq, S.; Kothari, S.L.; Kachhwaha, S. Seed Priming with Carbon Nanotubes and Silicon Dioxide Nanoparticles Influence Agronomic Traits of Indian Mustard (Brassica Juncea) in Field Experiments. *Journal of King Saud University - Science* **2022**, *34*, 102067, doi:10.1016/j.jksus.2022.102067.
134. Wang, W.; Zhang, C.; Zheng, W.; Lv, H.; Li, J.; Liang, B.; Zhou, W. Seed Priming with Protein Hydrolysate Promotes Seed Germination via Reserve Mobilization, Osmolyte Accumulation

and Antioxidant Systems under PEG-Induced Drought Stress. *Plant Cell Rep* **2022**, *41*, 2173–2186, doi:10.1007/s00299-022-02914-6.
135. Basit, F.; Liu, J.; An, J.; Chen, M.; He, C.; Zhu, X.; Li, Z.; Hu, J.; Guan, Y. Seed Priming with Brassinosteroids Alleviates Aluminum Toxicity in Rice via Improving Antioxidant Defense System and Suppressing Aluminum Uptake. *Environ Sci Pollut Res* **2022**, *29*, 10183–10197, doi:10.1007/s11356-021-16209-y.
136. ElSayed, A.I.; Rafudeen, M.S.; Ganie, S.A.; Hossain, M.S.; Gomaa, A.M. Seed Priming with Cypress Leaf Extract Enhances Photosynthesis and Antioxidative Defense in Zucchini Seedlings under Salt Stress. *Scientia Horticulturae* **2022**, *293*, 110707, doi:10.1016/j.scienta.2021.110707.
137. Hu, F.; Jiang, S.; Wang, Z.; Hu, K.; Xie, Y.; Zhou, L.; Zhu, J.; Xing, D.; Du, B. Seed Priming with Selenium: Effects on Germination, Seedling Growth, Biochemical Attributes, and Grain Yield in Rice Growing under Flooding Conditions. *Plant Direct* **2022**, *6*, e378, doi:10.1002/pld3.378.
138. Iqbal, W.; Afridi, M.Z.; Jamal, A.; Mihoub, A.; Saeed, M.F.; Székely, Á.; Zia, A.; Khan, M.A.; Jarma-Orozco, A.; Pompelli, M.F. Canola Seed Priming and Its Effect on Gas Exchange, Chlorophyll Photobleaching, and Enzymatic Activities in Response to Salt Stress. *Sustainability* **2022**, *14*, 9377, doi:10.3390/su14159377.
139. Sadeghizadeh, M.; Zarea, M.J. Effects of Seed Priming with Zinc on Germination, Nursery Seedling Growth and Paddy Fields Yield of Two Rice (Oryza Sativa L.) Cultivars. *J. Crop Sci. Biotechnol.* **2022**, *25*, 313–324, doi:10.1007/s12892-021-00133-1.
140. Noori, H.; Moosavi, S.G.; Rostampour, M.F. Responses of Cumin (Cuminum Cyminum L.) to Different Seed Priming Methods under Osmotic Stress. . *Not Bot Horti Agrobo* **2022**.
141. Kaushik, S.; Sharma, P.; Kaur, G.; Singh, A.K.; Al-Misned, F.A.; Shafik, H.M.; Sirhindi, G. Seed Priming with Methyl Jasmonate Mitigates Copper and Cadmium Toxicity by Modifying Biochemical Attributes and Antioxidants in Cajanus Cajan. *Saudi Journal of Biological Sciences* **2022**, *29*, 721–729, doi:10.1016/j.sjbs.2021.12.014.
142. Sharma, P.; Gautam, A.; Kumar, V.; Guleria, P. MgO Nanoparticles Mediated Seed Priming Inhibits the Growth of Lentil (Lens Culinaris). *Vegetos* **2022**, *35*, 1128–1141, doi:10.1007/s42535-022-00400-8.
143. Silva, P.C.C.; Azevedo Neto, A.D.; Gheyi, H.R.; Ribas, R.F.; Silva, C.R.R.; Cova, A.M.W. Seed Priming with H_2O_2 Improves Photosynthetic Efficiency and Biomass Production in Sunflower Plants under Salt Stress. *Arid Land Research and Management* **2022**, *36*, 283–297, doi:10.1080/15324982.2021.1994482.
144. Idrees, H.; Shabbir, I.; Khurshid, H.; Khurshid, A.; Tahira, R.; Fatima, F.; Younas, A.; Abbas, H. Seed Priming of Wheat Through Salicylic Acid to Induce Salt Tolerance. *Biol Clin Sci Res J* **2022**, *2022*, doi:10.54112/bcsrj.v2022i1.95.
145. Waqas Mazhar, M.; Ishtiaq, M.; Hussain, I.; Parveen, A.; Hayat Bhatti, K.; Azeem, M.; Thind, S.; Ajaib, M.; Maqbool, M.; Sardar, T.; et al. Seed Nano-Priming with Zinc Oxide Nanoparticles in Rice Mitigates Drought and Enhances Agronomic Profile. *PLOS ONE* **2022**, *17*, e0264967, doi:10.1371/journal.pone.0264967.
146. Choudhury, S.; Moulick, D.; Mazumder, M.K.; Pattnaik, B.K.; Ghosh, D.; Vemireddy, L.R.; Aldhahrani, A.; Soliman, M.M.; Gaber, A.; Hossain, A. An In Vitro and In Silico Perspective Study of Seed Priming with Zinc on the Phytotoxicity and Accumulation Pattern of Arsenic in Rice Seedlings. *Antioxidants* **2022**, *11*, 1500, doi:10.3390/antiox11081500.
147. Al-Salama, Y. Effect of Seed Priming with ZnO Nanoparticles and Saline Irrigation Water in Yield and Nutrients Uptake by Wheat Plants. In Proceedings of the LAFOBA2; MDPI, June 16 2022; p. 37.

148. Hossinifarahi, M.; Moazen, H.A.; Amiri, A.; Jowkar, M.M.; Mottaghipisheh, J. Evaluation of Seed Priming and Culture Media to Improve the Germination Performance and Quality of Sweet Pepper and Eggplant Seedlings. **2022**.
149. Pradhan, N.; Moaharana, R.; Ranasingh, N.; Biswal, K.; Bordolui, S. Effect of Seed Priming on Different Physiological Parameters of Cowpea (Vigna Unguiculata L. Walp) Seeds Collected from Western Odisha. *The Pharma Innovation Journal* **2022**, *11*, 2338–2343.
150. Moyo, M.; Amoo, S.O.; Van Staden, J. Seed Priming with Smoke Water and Karrikin Improves Germination and Seedling Vigor of Brassica Napus under Varying Environmental Conditions. *Plant Growth Regul* **2022**, *97*, 315–326, doi:10.1007/s10725-022-00821-0.
151. Younas, H.S.; Abid, M.; Ashraf, M.; Shaaban, M. Seed Priming with Silicon and Chitosan for Alleviating Water Stress Effects in Maize (ZEA MAYS L.) by Improving Antioxidant Enzyme Activities, Water Status and Photosynthesis. *Journal of Plant Nutrition* **2022**, *45*, 2263–2276, doi:10.1080/01904167.2022.2046070.
152. Bourhim, M.R.; Cheto, S.; Qaddoury, A.; Hirich, A.; Ghoulam, C. Chemical Seed Priming with Zinc Sulfate Improves Quinoa Tolerance to Salinity at Germination Stage. In Proceedings of the LAFOBA2; MDPI, June 16 2022; p. 23.
153. Salih, E.G.I.; Zhou, G.; Muddathir, A.M.; Ibrahim, M.E.H.; Ahmed, N.E.; Adam Ali, A.Y.; Zhu, G.; Jiao, X.; Meng, T.; Ahmad, I. Effects of Seeds Priming with Plant Growth Regulators on Germination and Seedling Growth of Hargel (Solenostemma Argel (Del.) Hayne) under Salinity Stress. *PAK. J. BOT.* **2022**, *54*, doi:10.30848/PJB2022-5(20).
